# Activated Macrophages of Monocytic Origin Predominantly Express Proinflammatory Cytokine Genes, Whereas Kupffer Cells Predominantly Express Anti-Inflammatory Cytokine Genes

**DOI:** 10.1155/2019/3912142

**Published:** 2019-03-05

**Authors:** Anastasia Lokhonina, Andrey Elchaninov, Timur Fatkhudinov, Andrey Makarov, Irina Arutyunyan, Maria Grinberg, Valeria Glinkina, Viktor Surovtsev, Galina Bolshakova, Dmitry Goldshtein, Gennady Sukhikh

**Affiliations:** ^1^National Medical Research Center for Obstetrics, Gynecology and Perinatology Named after Academician V.I. Kulakov of Ministry of Healthcare of Russian Federation, 4 Oparina Street, Moscow 117997, Russia; ^2^Pirogov Russian National Research Medical University, Ministry of Healthcare of The Russian Federation, 1 Ostrovitianov Street, Moscow 117997, Russia; ^3^Peoples' Friendship University of Russia, 6 Miklukho-Maklaya Street, Moscow 117198, Russia; ^4^Scientific Research Institute of Human Morphology, 3 Tsurupa Street, Moscow 117418, Russia; ^5^Research Centre of Medical Genetics, 1 Moscvorechie, 115478 Moscow, Russia

## Abstract

In the central nervous system and in the liver, the macrophage populations are represented exclusively by descendants of the hematopoietic progenitor cells of the yolk sac. The reasons for such differential distribution of macrophages are not fully understood. We found that, as can be judged by corresponding changes in the expression of CD86 and CD163 markers, the transient macrophages of monocytic lineage are more sensitive to activating stimuli. The two macrophage populations have distinct patterns of gene expression, which is particularly noticeable for M1- and M2-associated genes. For instance, Kupffer cells more readily develop and longer maintain the elevated expression levels of* Il4*,* Il10*, and* Il13* upon the activation; by contrast, the macrophages of monocytic lineage express* Il1b*,* Il12a*, and* Tnfα *upon the activation. The obtained results allow us to conclude that the* in vitro* activated Kupffer cells of the liver are committed to M2 phenotype, whereas the* in vitro* activated monocyte-derived macrophages show a typical M1 behavior. These observations are likely to reflect the situation in the* in vivo* microenvironments.

## 1. Introduction

Macrophages are the key regulatory participants of various morphogenetic processes in the mammalian body, particularly during the inflammation and tissue repair [1–4].

In accordance with contemporary concepts, macrophages originate from three sources, which correspond to three developmental generations of hematopoietic progenitor cells. The first of them, c-Kit^lo^CD41^lo^, are progenitor cells of the yolk sac [5, 6], which give rise to microglia of the central nervous system [7]. The second generation, c-Myb^+^CD45^lo^, are erythro-myeloid progenitor cells of the yolk sac [7], which migrate to the liver. The third generation of hematopoietic progenitor/stem cells, c-Kit^+^CD45^+^, originate from the aortal-gonadal-mesonephric area and gradually colonize the liver and the red bone marrow of an embryo [5, 6].

Macrophage populations of most organs in the prenatal period consist of the lineage from the second and third generations of hematopoietic cells (respectively, c-Myb^+^ CD45^lo^ and c-Kit^+^CD45^+^). However, this state is followed by a gradual decrease in the proportion of macrophages developing from the erythro-myeloid progenitor cells of the yolk sac and an increase in the proportion of macrophages developing from hematopoietic cells of the third generation [2, 5, 6]. These trends take place ubiquitously with the exclusions of the central nervous system, the liver, and the epidermis. Of these locations, the central nervous system is apparently locked for the immigration of the formed elements, whereas the liver and the epidermis normally harbor the self-sustained proliferating macrophage populations. These are derived from the second generation of hematopoietic cells and represented by Kupffer cells (KCs) in the liver and Langerhans cells in the epidermis [5, 6].

The source of origin is probably reflected in specific properties of organ macrophages, as well as in their attitude to regulation of physiological and pathological processes. At normal conditions, the liver is home to more than 95% of all macrophages of the body [8]. Nevertheless, at specific pathological conditions, e.g., after the acute hepatic injury induced with paracetamol or carbon tetrachloride, the liver is effectively colonized by monocytes, which obviously has a lot to do with the subsequent rescuing of the damaged organ [9, 10].

In this case, the arriving macrophages of bone marrow origin remain the dominant population only for the first 72 h after the intervention. At 96 h, they are totally eliminated and replaced by KCs, which by this time have already restored their numbers by means of proliferation [9]. Deliberate depletion of the resident macrophage population, as well as blocking the arrival of blood monocytes, significantly undermines the regeneration process [10, 11]. It is obvious that both types of macrophages, the resident self-sustaining KCs and the newly-arriving macrophages of monocytic lineage (MNCs), participate in the regeneration; however, their particular roles in this process remain understudied.

In accordance with the concept of the tissue niche as a major factor of macrophage differentiation, the differences in the properties of macrophages are determined not so much by their source of origin, but predominantly by the niche, that is, their immediate tissue microenvironment [12, 13].

The concept of niches is biologically consistent and useful from the point of view of studying the functioning of macrophages within particular tissue types; however, this concept leaves unexplained certain important phenomena associated with the compensatory growth and regeneration of the liver. First of all, this concept implies a gradual increase in the proportion of bone marrow macrophages as the liver grows. However, the proportion of bone marrow macrophages in the liver of rats and mice remains approximately the same. After birth, the proportion of monocytic macrophages in the liver of mice reaches about 2-5% and subsequently stabilizes at that level [5, 6]. Besides, it is not clear why, after the APAP-induced toxic liver injury, the macrophages of monocytic origin are not accumulated in the liver, but almost completely eliminated from there, whereas the population of resident macrophages, Kupffer cells, is restored [10].

Thus, the properties of macrophages are determined by both the microenvironment of macrophages and the source of their origin.

The current comparative study aims to estimate similarity between the naïve and activated MNCs and KCs in the terms of immune phenotypes, as well as cytokine gene expression profiles and phagocytic activities. In the present study, we obtain direct evidence that the properties of macrophages are determined not only by tissue microenvironments, but also by the source of origin.

## 2. Materials and Methods

### 2.1. Animals

The experiments were done on the outbred Wistar male rats of 250±20 g body weight (n=50), 5 weeks old, obtained from the facilities of Shemyakin-Ovchinnikov Institute of Bioorganic Chemistry RAS (Pushchino, Moscow region). Animal care and the experiments were carried out in accordance with the order of the Ministry of Health of the USSR No. 755, 12/08/1977, and the European Convention for the Protection of Vertebrate Animals, Strasbourg, 18/03/1986. All experimental work involving animals was carried out according to the standards of laboratory practice authorized by National Guidelines No. 267 by Ministry of Healthcare of the Russian Federation, 01/06/2003, and all efforts were made to minimize suffering. The permit on this study was granted by Bioethics Committee of the Scientific Research Institute of Human Morphology, Record No. 5, 19/01/2017.

The animals, two per cage, were housed in a temperature-regulated room with a 12:12 h light-dark cycle and unlimited access to food and water.

### 2.2. Isolation of Blood Monocytes

The rats were deeply anesthetized with diethyl ether (Medhimprom, Moscow region, Russia; 0.08 ml per liter of chamber volume) to collect blood by cardiac puncture. The collected blood was mixed 1:1 with HBSS (Hanks Balanced Salt Solution) supplemented with heparin (1000 U/ml, Sintez, Russia). The fraction of mononuclear cells was separated by density gradient centrifugation with Ficoll (PanEco, Russia) at 400 g and 20°С for 30 min. The cells were washed twice with HBSS at 300 g and 20°С for 20 min. Cell numbers and viability were assessed with a TC20 Cell Counter (Bio-Rad, USA).

### 2.3. Isolation of KCs from the Liver

The rats were deeply anesthetized with diethyl ether (Medhimprom, Moscow region, Russia; 0.08 ml per liter of chamber volume) for liver perfusion with 40-50 ml PBS via portal vein. The liver was excised, washed twice with HBSS, cleared from membranes and large vessels, minced, and incubated in 0.05% solution of collagenases I and IV (PanEco) for 20 min at 37°С in an orbital shaker. The resulting cell suspension was passed through a 100 *μ*m nylon filter (SPL LifeScience, Korea) and washed twice in HBSS at 300 g and 20°С for 20 min. The pellets were resuspended in 30 ml of PBS; parenchymal cells of the liver were subsequently discarded by sedimentation at 50 g and 20°С for 3 min. The supernatant with nonparenchymal cells was subjected to gradient centrifugation at 400 g and 20°С for 30 min with Ficoll (PanEco) to obtain the desired fraction of KCs [14, 15]. Cell numbers and viability were assessed with a TC20 Cell Counter (Bio-Rad).

### 2.4. Cell Culture of Macrophages

The obtained rat KCs and blood monocytes were transferred to RPMI medium (PanEco) supplemented with 10% fetal calf serum (PAA Lab, Austria) and 1% penicillin-streptomycin (PanEco). The medium was changed on day 2, and all the unattached cells were removed; half-volumes of the culture medium were replaced with the fresh portions on days 4 and 7.

### 2.5. Activation of Macrophages toward M1- and M2-Phenotypes

For the M1 type of macrophage activation, KCs and MNCs were separately cultured in RPMI medium (PanEco) supplemented with 10% fetal calf serum, 1% penicillin-streptomycin, and 50 ng/ml GM-CSF (Cloud-Clone Corp, USA). The medium was changed on day 2 and all the unattached cells were removed; half-volumes of the culture medium were replaced with the fresh portions on days 4 and 7. The M1 activation was induced on day 7 by introducing LPS (Sigma-Aldrich, USA) and IFN-*ɣ* (Cloud-Clone Corp) to final concentrations of 40 ng/ml each (M1-medium).

For the M2 type of macrophage activation, KCs and MNCs were separately cultured in RPMI medium (PanEco) supplemented with 10% fetal calf serum, 1% penicillin-streptomycin, and 100 ng/ml M-CSF (Cloud-Clone Corp.). The medium was changed on day 2, and all the unattached cells were removed; half-volumes of the culture medium were replaced with the fresh portions on days 4 and 7. The M2 activation was induced on day 7 by introducing IL4, IL10, and IL13 (Cloud-Clone Corp.) to final concentrations of 20 ng/ml (M2-medium).

For the control, KCs and MNCs were separately cultured in RPMI medium supplemented with 10% fetal calf serum and 1% penicillin-streptomycin without inducers and were conventionally considered as М0 macrophages.

### 2.6. Macrophage Marker Expression

The measurements were made at 24 h after the macrophage activation. Expression of distinctive proteins of the activated macrophages was evaluated using antibodies to CD68 (1:100, Abcam, UK), iNOs (1:100, Abcam), Arg1 (1:100, Abcam), and CD206 (1:100, Santa Cruz, USA), in combination with FITC-conjugated secondary antibodies (1:200, Abcam); the nuclei were counterstained with DAPI (Sigma-Aldrich). The evaluation was carried out by using a Leica DM 4000 B fluorescent microscope and LAS AF v.3.1.0 build 8587 software (Leica Microsystems, Germany).

The total number of cells and the number of cells positively stained with the corresponding antibody were counted in the slides. The index reflecting the proportion of positive cells was calculated as the ratio of stained cells to the total number of cells. For each point, not less than 100 cells were counted. The index is expressed in %.

### 2.7. Flow Cytometry

The samples were collected at 24 h after the macrophage activation. The cells were detached from the support with Trypsin-Versene and washed twice with HBSS. Cell permeabilization and fixation for subsequent immunostaining of intracellular markers were carried out by using the Inside Stain Kit (Miltenyi Biotec, Germany) in accordance with the manufacturer's recommendations. For cell surface immunostaining, the cells were resuspended in PBS (100×10^3^ cells in 100 *μ*l). The antibodies used in the study included CD45-PerCPVio700, CD68-PEVio770 (Miltenyi Biotec), CD86-VioBright FITC (Miltenyi Biotec), CD11b-PE (Miltenyi Biotec), and CD163-PE (Thermo Fisher, USA). The assay was carried out by using a Cytomics FC 500 flow cytometer with CXP software (Beckman Coulter, USA).

### 2.8. Quantitative PCR

The samples were collected at 24 and 72 h after the macrophage activation. The cell suspensions were immediately fixed with RNAlater RNA Stabilization Reagent (QIAGEN, Germany) and kept at +4°С for 1 day, then transferred to a low temperature freezer, and stored at -80°С. The total RNA was isolated with RNeasy Plus Mini Kit (QIAGEN). Synthesis of cDNA of the total RNA templates was carried out with MMLV RT kits (Evrogen, Russia). The obtained cDNA samples were subsequently analyzed by quantitative real-time PCR with qPCRmix-HS SYBR Green (Evrogen, Russia). The oligonucleotide sequences were designed by using mRNA and genomic sequences available from NCBI Nucleotide data base and NCBI Primer-BLAST software in accordance with the general rules of primer design; the oligos (Table 1) were ordered from Evrogen. The fluorescence readouts were automatically quantitated by using the Ct approach; the values were normalized in accordance with Pfaffl algorithm [16] with* Gapdh* as a reference target. To demonstrate the differences between the experimental and control groups more clearly, the expression of a particular gene in M0 macrophages of monocytic origin or Kupffer cells was taken as 1. The levels of gene expression in activated macrophages were compared with corresponding values for the nonactivated cells.

### 2.9. ELISA of Cytokine Production

Quantitative measurements of TNF-*α*, Il1b, Il6, and Il10 protein concentrations in conditioned media were carried out using ELISA kits by Cloud-Clone Corp. in accordance with the manufacturers protocols. The measurements were made for the media collected on days 1 and 3 after the onset of activation.

### 2.10. Evaluation of Microsphere Phagocytosis

For the real-time video recording of phagocytic activity, the macrophages were plated in round dishes with 170 *μ*m thick bottoms and optical refractive index of 1.52 (Ibidi, Germany). The macrophages were subsequently activated as described in Section 2.5 and exposed to 1.5 *μ*m latex beads (Dia-m, Moscow, Russia) by addition of 5 *μ*l of 10% suspension of the beads per 1 ml of the medium. The imaging of living cells (phase contrast microscopy, ×400) at 1, 2, 5, and 24 h after the addition of latex beads was carried out using an inverted microscope Zeiss Axiovert 40 CFL with AxioVs40 4.8.2.0 software. A number of beads per cell were evaluated by using the Adobe Photoshop CS6 software for counting all cells in 10 randomly selected fields.

### 2.11. Statistics

The data were analyzed with SigmaStat 3.5 program package (Systat Software Inc, USA). Sample proportions were compared by 2-sample z-test; relative gene expression values were compared by the Mann-Whitney U test; more-than-two-groups comparisons were done using ANOVA on ranks;* p*<0.05 for the differences were considered statistically significant.

## 3. Results

### 3.1. Comparative Analysis of Surface Marker Phenotypes

**Figure 1:**
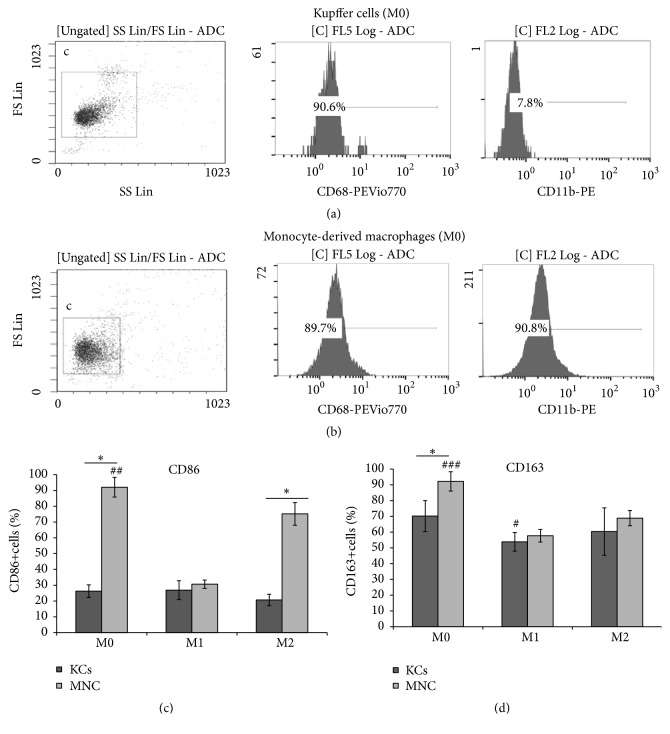
Flow cytometry assessment of surface phenotypes for MNCs and KCs, nonactivated and activated in either M1- or M2-direction. (a) Flow cytometry assessment of surface phenotypes for nonactivated KCs, (b) flow cytometry assessment of surface phenotypes for nonactivated MNCs, (c) flow cytometry assessment of CD86 marker for MNCs and KCs, nonactivated and activated in either M1- or M2-direction, and (d) flow cytometry assessment of CD163 marker for MNCs and KCs, nonactivated and activated in either M1- or M2-direction. *∗p* < 0.05 MNCs (M0) versus KCs (M0), CD86^+^MNCs (M2) versus CD86^+^KCs (M2), #*p* < 0.05 CD163^+^KCs (M0) versus CD163^+^KCs (M1), ##* p* < 0.05 CD86^+^ MNCs (M0) versus CD86^+^ MNCs (M1) and CD86^+^ MNCs (M2), and ###* p* < 0.05 CD163^+^ MNCs (M0) versus CD86^+^ MNCs (M1) and CD86^+^ MNCs (M2); KCs: Kupffer cells; MNCs: macrophages of monocytic lineage.

**Figure 2:**
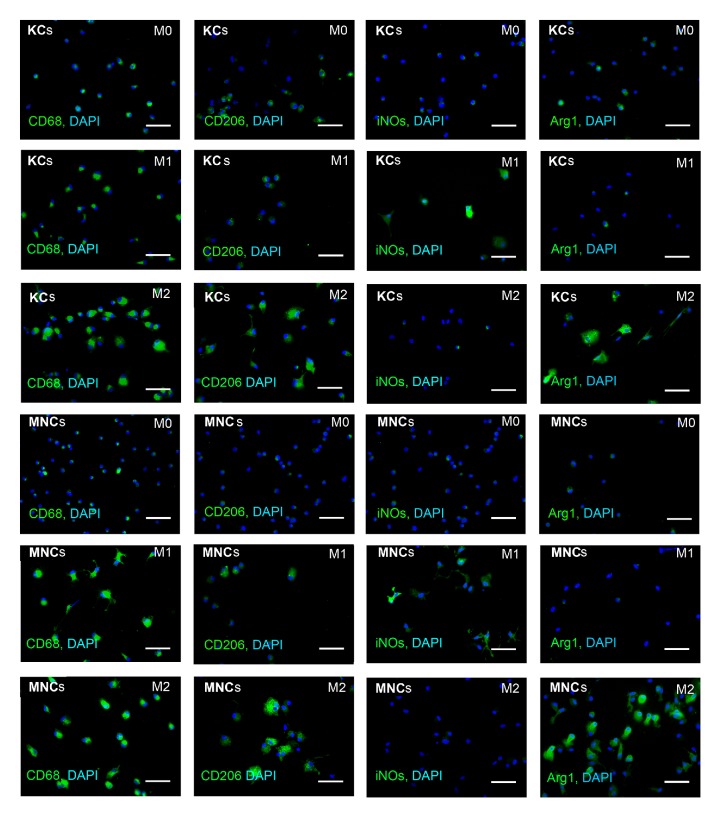
Immunocytochemical assessment of surface phenotypes for KCs and MNCs under the influence of M1 or M2 inducers. Fluorescent microscopy images display immunostaining (FITC, green); cell nuclei are counterstained with DAPI (blue); KCs: Kupffer cells; MNCs: macrophages of monocytic lineage; scale bar, 50 *μ*m.

**Figure 3:**
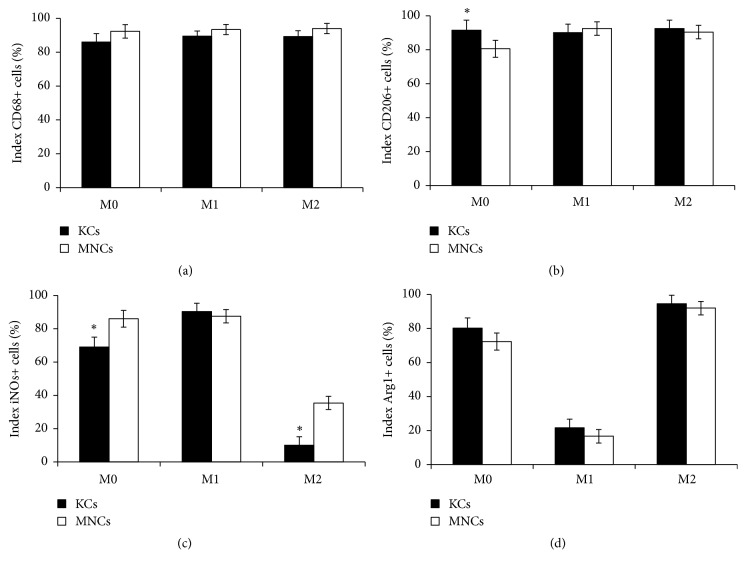
Quantification of immunocytochemical assessment of phenotypes for KCs and MNCs under the influence of M1 or M2 inducers. The asterisks indicate significant differences from the corresponding values,* p*<0.05; KCs: Kupffer cells; MNCs: macrophages of monocytic lineage.

The obtained cultures of MNCs and KCs had similar high proportions of CD68^+^ cells, about 90%, which indicated high selectivity of the isolation procedure (Figures 1(a), 2, and 3). However, the macrophages isolated from the liver expressed CD11b at low level, whereas the majority of the nonactivated cultured MNCs had the CD11b^hi^ phenotype (Figures 1(a) and 1(b)). In addition, KCs expressed CD206 at a significantly higher level as compared with the macrophages of monocytic origin, whereas the nonactivated cultured MNCs expressed iNOs at a significantly higher level (Figures 2, 3(b) and 3(c)).

Of the nonactivated M0 cells, the cultured MNCs predominantly showed CD86 expression (with more than 90% of CD86^+^ cells), significantly differing in these characteristics from KCs (with about 26% of CD86^+^ cells) (Figure 1(c)).

The activating factors (inducers) had no significant influence on CD86 and CD163 expression in KCs (Figures 1(c) and 1(d)).

Under the influence of М1 activating medium, the frequency of CD86 expression in cultured MNCs significantly decreased, whereas the М2 activating medium supported CD86 expression at high levels as compared with M0 (Figure 1(c)).

CD163 was expressed at high levels by all M0 cultures, both MNCs and KCs; however, the exact proportion of CD163^+^ cells in M0 MNCs was significantly higher. Under the influence of M1 and M2 activating factors, the numbers of CD163^+^ MNCs significantly decreased (as compared with M0 MNCs) to the levels comparable with KCs (Figure 1(d)).

Comparative immunocytochemical examination of cell surface antigens expressed by MNCs and KCs showed major similarity in their responses to the inducing stimuli. Under the influence of M1 media, MNCs and KCs started to form processes; they unevenly expressed the induced NO synthase and did not express arginase (Figures 2, 3(c) and 3(d)). Under the influence of M2 media, the macrophages retained the round shape, expressed both CD206 and arginase, and did not express the induced NO synthase (Figures 2, 3(c) and 3(d)), which fit well with the data on expression of corresponding genes,* iNOs* and* Arg1*, for the induced NO synthase and arginase, respectively. Neither M1 nor M2 media influenced the expression of CD68 (Figures 2 and 3(a)). Under the influence of M2 media the expression level of iNOs was significantly higher in macrophages of monocytic origin than in Kupffer cells (Figures 2 and 3(c)).

### 3.2. Gene Expression Analysis

**Figure 4:**
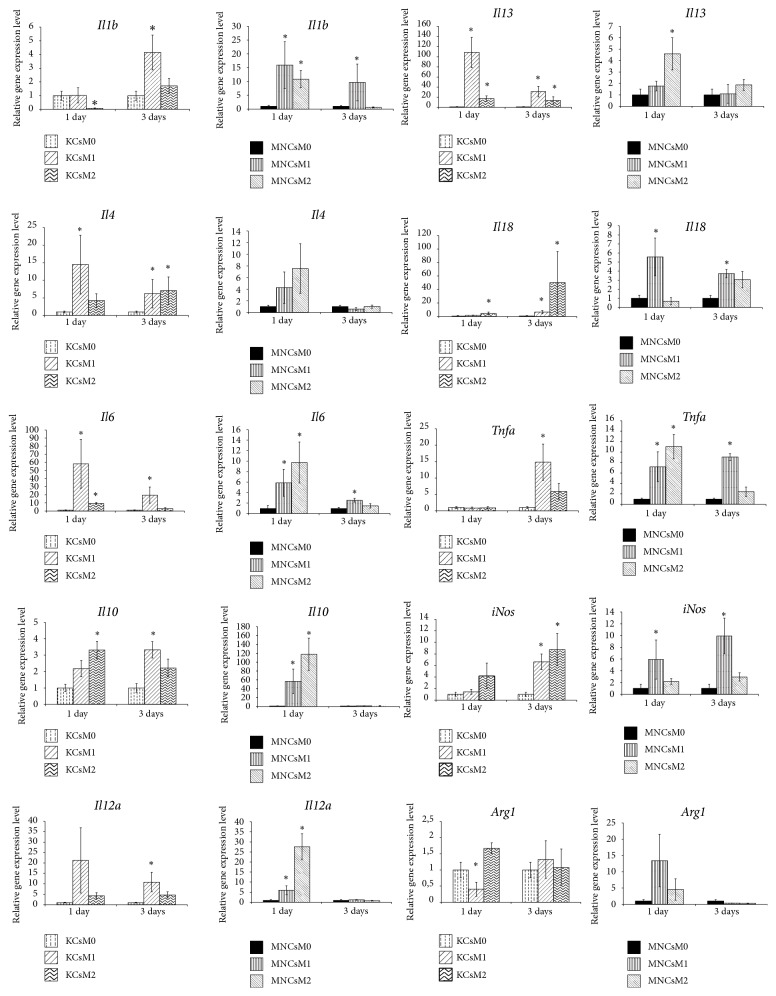
Gene expression profiles for cultured MNCs and KCs in different states (nonactivated and either M1- or M2-induced at consecutive time points of 1 and 3 days after the onset of activation). The data is presented as means with the bars for standard deviations. Horizontal axes represent time after the onset; vertical axes represent expression level in relative units. The asterisks indicate significant differences from the corresponding values for the nonactivated M0 macrophages;* p*<0.05; KCs: Kupffer cells; MNCs: macrophages of monocytic lineage.

Analysis of representation for distinctive mRNA targets in the studied cell cultures (Figure 4) allowed us to find the difference between MNC and KC responses to the M1 and M2 inducing stimuli.

As compared with KCs, the cells of monocytic origin more readily responded to both types of inducers by switching on the proinflammatory cytokine-encoding genes, and their response lasted longer. For instance, significant increases in expression of* Il1b* and* Tnfα* by MNCs were observed for both M1 and M2 activation, of which M1 activation resulted in a longer response than M2 (Figure 4). In KCs, the expression of* Tnfa* significantly increased as late as on day 3 after the onset of M1 activation, whereas no significant changes in* Il1b* expression by KCs upon the activation were detected in these experiments (Figure 4).

Expression of* Il6* by the activated MNC and KC cultures showed similar dynamics, with a rapid significant increase on day 1; however, this effect was sustainable and lasted 3 days for M1 only. Expression of* Il12a* by MNCs was rapidly upregulated by both M1 and M2 inducers on day 1, whereas a similar response by KCs took 3 days to develop for M1, and no changes in* Il12a* expression by KCs were observed in the case of M2. It should be noted that nonactivated M0 MNCs expressed both* Il6* and* Il12a* genes significantly stronger than nonactivated M0 KCs (Figure 4).

Expression of* Il18* by MNCs was significantly upregulated with M1, but not M2, activation, whereas the expression of* Il18 *by KCs was increased under both types of activation.

A similar analysis for the anti-inflammatory cytokine-encoding genes showed that these genes were more readily upregulated in KCs than in MNCs; the elevated levels of their expression in KCs were also more sustainable. For instance, a significant increase in the expression of* Il4* was observed in KCs only. In particular, the elevated levels of* Il4* expression by KCs were detected on day 1 for M1 activation and on day 3 for both M1 and M2 activation. Similarly, expression of* Il13* in KCs was elevated on days 1 and 3 for both types of activation, whereas its expression in the MNCs was only transiently increased under the influence of M2 inducers (Figure 4).

The influence of inducing stimuli led to upregulation of* Il10 *expression in both types of cultured macrophages. In KCs, the elevated levels of its expression were observed on day 1 of M2 induction and on day 3 of M1 induction. In MNCs, the elevated expression levels of* Il10* were observed on day 1 of M1 induction only.

The influence of M1 macrophage activation medium caused a prolonged elevation of* iNOs* expression in MNCs, detectable on days 1 and 3 of the activation, whereas no changes in* iNOs* expression were observed in the course of M2 activation. In KCs, an increase in* iNOs* expression was observed on day 3 of activation only; its expression was significantly increased in both M1 and M2 activating media (Figure 4).

Expression level of the arginase-encoding gene* Arg1* in the nonactivated M0 KCs was significantly higher as compared with the counterpart M0 MNC culture. On day 1 of activation, its expression in KCs was significantly stronger induced for M2 than for M1 (Figure 4).

### 3.3. Cytokine Production Assay

**Figure 5:**
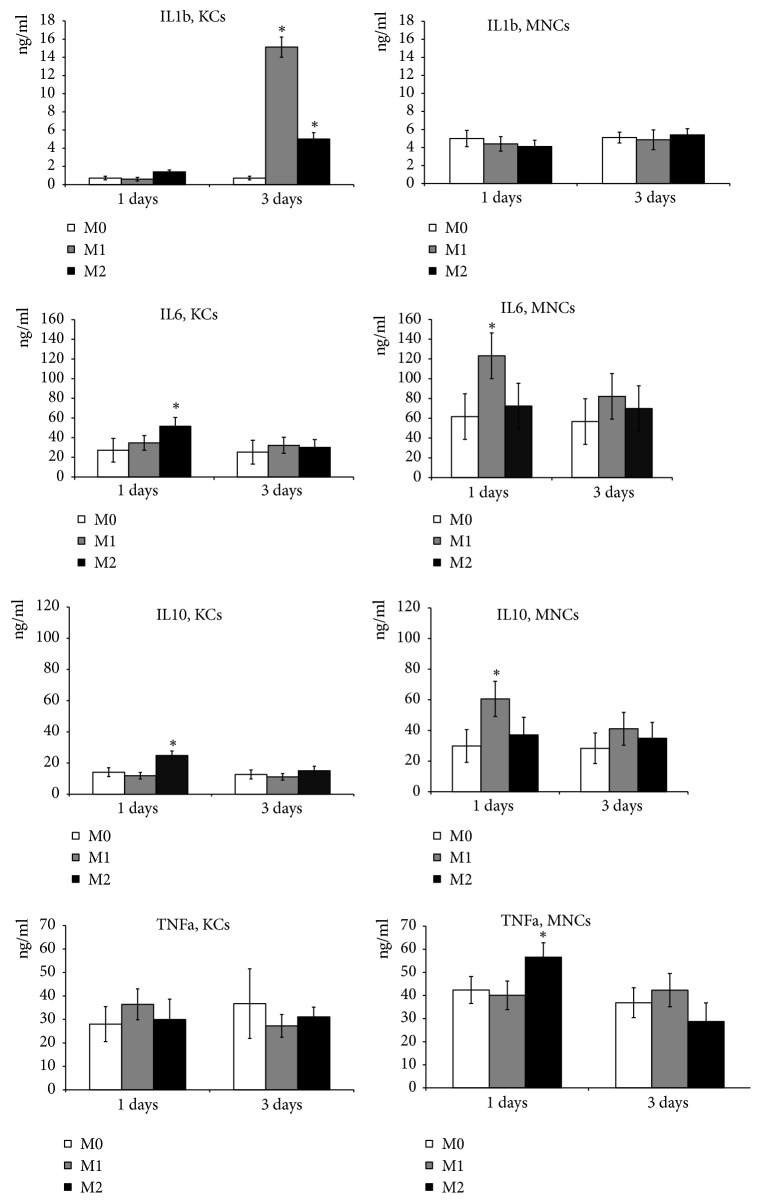
Dynamics of cytokine production by cultured MNCs and KCs. The vertical axis represents concentration, ng/ml. KCs: Kupffer cells; MNCs: macrophages of monocytic lineage. The asterisks indicate significant differences from the corresponding values for the nonactivated M0 macrophages,* p*<0.05.

The measurement of IL1b concentrations in the culture medium indicated that its secretion by KCs was significantly increased on day 3 of M1 and M2 induction. Secretion of IL 1b by MNCs showed no changes in response to either type of the induction (Figure 5).

The dynamic changes in secreted cytokine production for IL 6 and IL 10 were similar in character. Concentrations of these cytokines in KC and MNC cultures were significantly increased by day 1 of the induction, however, with the opposite specificity: KCs specifically responded to M2 inducers, whereas MNCs responded to M1 inducers (Figure 5).

Production of TNF*α* by KCs and MNCs under the given experimental conditions differed. In particular, concentrations of TNF*α* in the KC-conditioned media after M1 and M2 induction were similar, whereas concentration of TNF*α* in the MNC-conditioned media was significantly and specifically increased on day 1 of M2 induction (Figure 5).

### 3.4. Microsphere Phagocytosis Assay

**Figure 6:**
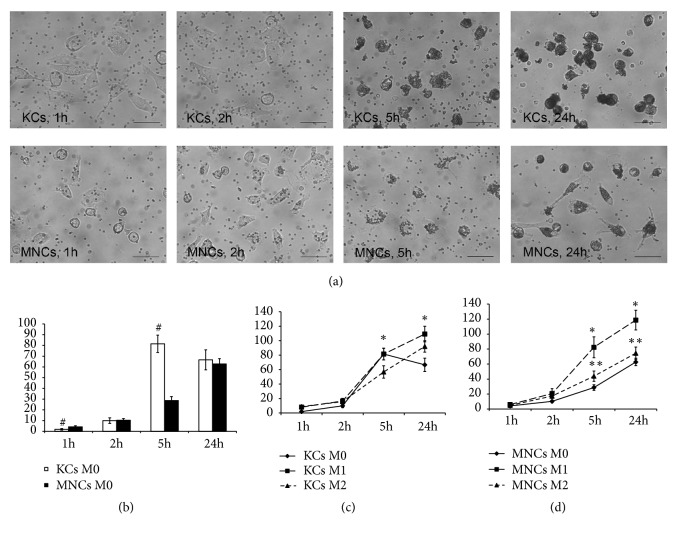
Microsphere phagocytosis assay. (a) Phagocytosis of nonactivated (M0) Kupffer cells and macrophages of monocytic origin, phase contrast, scale bar, 50 *μ*m; (b) graph representing phagocytosis assay of nonactivated macrophages; (c) graph representing phagocytosis assay of nonactivated and activated in either M1- or M2-direction for KCs, (d) graph representing phagocytosis assay of nonactivated and activated in either M1- or M2-direction for MNCs; horizontal axes represent time after the onset; vertical axes represent number of particles per cell; #: significant differences between nonactivated KCs and MNCs,* p*<0.05; *∗*: significant differences between macrophages under the influence of M1 inducers and nonactivated KCs and MNCs; *∗∗*: significant differences between macrophages under the influence of M2 inducers and nonactivated KCs and MNCs; KCs: Kupffer cells; MNCs: macrophages of monocytic lineage.

Comparative assay of phagocytic activity of the nonactivated M0 macrophages from different sources showed that MNCs were significantly more active, as indicated by the clearance rates of latex particles, according to the measurements made at 1 h after the addition of the particles to the culture medium (Figures 6(a) and 6(b)). A subsequent increase in phagocytic activity of KCs resulted in the lack of difference in phagocytic activity between KCs and MNCs at 2 h after the addition of the particles to the culture medium. Furthermore, at 5 h of the observation the rates of phagocytosis by KCs substantially exceeded the corresponding activity of MNCs; by 24 h of the observation the difference in phagocytic activity between the cultures completely ceased (Figures 6(a) and 6(b)).

Under the influence of M1 and M2 inducers, the phagocytic activity of macrophages increased in all of the examined cultures. The observed phagocytosis-stimulating influence of M1 inducers was significantly stronger, as compared with M2. It should be noted that MNCs were more susceptible to this effect of M1 activation, as their phagocytic activity remained significantly elevated, as compared with the nonactivated M0 MNCs, as late as at 5 h after the addition of the particles. The cultures of KCs, by contrast, showed no corresponding difference between the M1-activated and the M0 cells at 5 h after the addition of the particles (Figures 6(c) and 6(d)).

## 4. Discussion

Macrophages are considered very important participants in the regulation of repair processes, as well as in the development of pathological conditions in various organs including the liver [8, 11].

The currently predominant concept of M1/M2 macrophage polarization reflects the intrinsic functional plasticity of macrophages. It is generally accepted that the M1 polarized macrophages (that is, macrophages activated towards M1 type of molecular profiles and functional behaviors) release a number of proinflammatory cytokines and regulate repair processes at the early stage, whereas the M2 polarized macrophages exert anti-inflammatory action and regulate repair processes at their final stages. However, the most recent ideas in the field of macrophage research are dealing with a continuous range of functional states, of which the pure M1 and M2 states are just the extremes. Pure M1 or M2 macrophage populations are unachievable even under the action of specific inducers* in vitro*, because the cells inevitably interact with the xenogeneic proteins of the culture medium and the polymer material of the support [17].

The source of origin of any given macrophage is also relevant to the mode of its activation and functioning in health and pathology [2, 3]. Functional comparison of macrophages derived from the embryonic hematopoietic progenitor sources, exemplified by KCs, with macrophages of bone marrow origin, carried out in this study, allows us to make several conclusions.

In this study we have shown that the macrophages derived from the embryonic hematopoietic progenitor sources and the macrophages of bone marrow origin, which differentiate from monocytes of the blood, are unequal in their sensitivity to activating inducers. Certain indirect support to this finding can be found in the literature [18]. Particular results of the current study, which are indicative of the differential sensitivity of macrophages depending on their source of origin, deal with differential functioning of these cells under specific influence of M1 and M2 polarization inducers. However, the nonpolarized M0 macrophages isolated from different sources initially differ by their surface protein signatures. For instance, Kupffer cells of the liver were almost clear from CD11b integrin and showed poor surface expression of CD86, whereas the majority of nonpolarized macrophages of bone marrow origin were distinctly CD11b^+^CD86^+^.

In our experiments, Kupffer cells showed invariable surface expression of CD86, the protein which plays a key role in activation, proliferation, cytokine production, and differentiation of T cells [19], and CD163, which participates in the recognition of bacterial antigens and regulates the onset of local inflammatory reactions [20]. By contrast, surface expression of these markers by macrophages of bone marrow origin was subject to significant dynamics depending on the presence of inducers. Rather unexpectedly, surface expression of CD86 by the bone marrow-derived macrophage cultures was stronger suppressed by M1 than by M2 inducers; at the same time, a decrease in surface expression of CD163 by the bone marrow-derived macrophage cultures was detected for both types of inducers.

Somewhat controversial related data can be found in the literature, obtained on the well-established research model of repopulation of the liver by bone marrow-derived macrophages in the aftermath of the ionizing irradiation treatment. High doses of lipopolysaccharide (LPS) are shown to reduce the expression of CD163-encoding gene in resident Kupffer cells, but not the migrating bone marrow-derived macrophages [18]. The apparent discrepancy with our results can be explained by striking difference between the strong inducing stimulation with 100 mg/g LPS using the* in vivo* mouse model and the* in vitro* stimulation of isolated rat macrophages with relatively low doses of LPS (at 40 ng/ml of culture medium).

We showed that distinct groups of genes with inducible expression were specifically activated in cultured macrophages derived from different sources. We found that, independently of the state of activation or the influence of polarizing inducers, the macrophages of bone marrow origin were prone to more rapid and more sustainable upregulation of proinflammatory cytokine-encoding genes* Il1b*,* Il12a*, and* Tnfα*, whereas Kupffer cells more readily expressed the anti-inflammatory* Il4*,* Il10*, and* Il13. *It should be noted that initial levels of* Il6* и* Il12a* proinflammatory cytokine gene expression in the bone marrow-derived cultured macrophages were significantly higher than in the counterpart nonactivated cultured Kupffer cells.

The reported* in vitro* studies indicate that the repeated treatment with LPS may result in development of LPS tolerance, as revealed by the missing upregulation of the LPS-dependent genes in response to the treatment [21]. The effect of LPS tolerance may be relevant to differential sensitivity of macrophages from different sources (e.g., bone marrow derived macrophages and Kupffer cells) to the polarization inducers in general and to LPS in particular. Endotoxin, which is another name for LPS, is predominantly produced in the colon and absorbed and primarily transported to the liver, where it constantly acts on Kupffer cells [22].

The observed late onset of the activation-induced proinflammatory cytokine-encoding gene expression in Kupffer cells, especially that of* Tnfα*, is probably indicative of certain LPS tolerance in the resident macrophages of the liver. The low-level production of Tnf*α* in the liver after subtotal hepatectomy, which provides a good model of a small for size liver remnant, may be due to the same effect of LPS tolerance [23, 24].

It should be noted that all the examined macrophage cultures, independently of their origin, showed upregulation of both pro- and anti-inflammatory gene expression and cytokine production in response to polarization-inducing stimuli; in general terms, such a response was similar for both types of inducers, M1 and M2. This result supports the hypothesis of phenotypical continuum of macrophage activation, with polar M1 and M2 states as range limits [17]. In addition, we experimentally showed that the macrophages of bone marrow origin were especially susceptible to M1 activation, upon which they react faster and stronger than Kupffer cells, whereas the Kupffer cells were apparently more predetermined towards M2 type of induction. These results are in good compliance with data, on the basis of which we conclude that macrophages of bone marrow origin are adapted to the functioning of the liver under conditions of inflammation, while Kupffer cells (resident macrophages) are adapted to the normally functioning liver [12, 13].

In the aspect of the Kupffer cell participation in various kinds of liver repair, the observed upregulation of* Il4* and* Il13* expression is particularly important, because the roles of corresponding IL4 and IL13 cytokines in tissue repair are still understudied. Solitary lines of evidence indicate that IL4 specifically participates in the onset of hepatocyte proliferation within regenerating liver [25], whereas IL13 regulates proliferation of cholangiocytes and activity of fibroblasts within the liver [26].

The cultured Kupffer cells, as opposed to the cultured macrophages of bone marrow origin, showed distinctive dynamics of phagocytic activity. In particular, the nonactivated macrophages of monocytic origin exhibited steady rates of endocytosis, whereas the cultured Kupffer cells showed the pronounced dynamics of phagocytic activity with a sharp increase at early stages followed by a subsequent sharp decrease. The addition of polarizing inducers of either type (M1 or M2) upregulated the phagocytic activity of macrophages independently of their origin.

Noteworthy, at 1 h after the addition of latex particles to the culture medium, the nonactivated macrophages of bone marrow origin were significantly more active phagocytes as compared with the Kupffer cells. This result is in good compliance with the reported observation of higher phagocytic activity of the migratory macrophages, which arrive in the liver from the blood in the aftermath of the ionizing irradiation treatment, as compared with phagocytic activity of the resident Kupffer cells [18].

Phagocytic activity and antigen presentation are the main functions of macrophages. Differential capabilities of these two functions are probably a key to understanding the distribution of macrophages from different hematopoietic sources in the mammalian body. This idea is strongly supported by the fact that loose connective tissue of the intestine, as well as the skin dermis, is populated exclusively by macrophages of bone marrow origin, which differentiate from monocytes of the blood [2, 5, 6].

We consider it necessary to point out some limitations in the interpretation of the data obtained. First, it is well known that Ficoll may have an adverse effect on the cells during their isolation by a gradient method. The use of Ficoll for gradient isolation of macrophages is a widely used technique. Even for cell isolation using magnetic sorting, it is often recommended cell separation on a Ficoll gradient as an initial step [27, 28]. However, we used a protocol that included the stage of predifferentiation of the isolated monocytes and Kupffer cells in the medium with M-CSF or GM-CSF for several days. This protocol allows to obtain differentiated macrophages of monocytic origin [29], reduces the negative impact of Ficoll on isolated cells, and results in macrophages with a more stable phenotype [27, 28].

Secondly, Kupffer cells are mature, differentiated macrophages whereas blood monocytes (immature macrophages) are terminally differentiated upon culture. Macrophages predifferentiation used in our study also makes to level out the conditions of the microenvironment in which the cells were located prior to their isolation and ultimately to reveal the difference between the macrophages of different embryonic origin.

In addition, M0 macrophages of monocytic origin and Kupffer cells expressed approximately the same level of a number of macrophages markers, such as CD68, CD163, and CD206, and did not differ in the expression level of cytokine genes, except for the* Il6* and* Il12a* genes, which were expressed at a higher level in macrophages of monocytic origin. These facts indicate that monocyte-derived macrophages and Kupffer cells in a nonactivated state had similar properties, despite the different initial state. Besides, it might be incorrect to say unequivocally that monocytes differentiated into macrophages during cultivation, while Kupffer cells were terminally differentiated cells from the beginning of the experiment. Monocytes are sometimes referred to as the resident blood tissue macrophages; they are highly specialized and differentiated cells [30].

## 5. Conclusions

Comparative study of the phenotype, gene expression profile, cytokine production, and phagocytic activity of cultured macrophages allowed us to reveal certain specific features of Kupffer cells as compared with the macrophages of bone marrow origin. The monocyte-derived macrophages turned out to be more sensitive to activating factors. Upon activation with М1 and М2 inducers, the cultured Kupffer cells showed more rapid and sustainable upregulation of the anti-inflammatory cytokine-encoding genes, whereas the monocyte-derived cultured macrophages showed more rapid and sustainable upregulation of the proinflammatory cytokine-encoding genes. It remains as yet unclear whether the observed specific features and behaviors of Kupffer cells are common to all macrophages derived from the embryonic hematopoietic progenitor sources, or these features just reflect the peculiarity of the liver microcirculatory system and the unique hepatic microenvironments. Figuring it out would require subsequent comparative functional investigation of macrophages from different developmental sources.

## Figures and Tables

**Table 1:** PCR primers.

Gene	Forward and reverse primer sequences
*Il1b*	CTG TCT GAC CCA TGT GAG CT
	ACT CCA CTT TGG TCT TGA CTT

*Il4*	ATG TAA CGA CAG CCC TCT GA
	AGC ACG GAG GTA CAT CAC G

*Il6*	TAC ATA TGT TCT CAG GGA GAT
	GGT AGA AAC GGA ACT CCA G

*Il10*	GCC CAG AAA TCA AGG AGC AT
	TGA GTG TCA CGT AGG CTT CTA

*Il12a*	CTG CCA AGT GTC TTA ACC AGT
	GCA GGC CTC CAG TGT GCT

*Il13*	CCA GAA GAC TTC CCT GTG CA
	CCC TCA GTG GCC ATA GCG

*Il18*	GAC AAA AGA AAC CCG CCT G
	ACA TCC TTC CAT CCT TCA CAG

*Tnf*	CCA CCA CGC TCT TCT GTC TA
	GCT ACG GGC TTG TCA CTC G

*iNos*	CGC TGG TTT GAA ACT TCT CAG
	GGC AAG CCA TGT CTG TGA C

*Arg1*	GGA TGA GCA TGA GCT CCA AG
	GCC AGC TGT TCA TTG GCT T

*Gapdh*	GCGAGATCCCGCTAACATCA
	CCCTTCCACGATGCCAAAGT

## Data Availability

The data used to support the findings of this study are available from the corresponding author upon request.

## Abbreviations

KC: Kupffer cell
MNC: Macrophage of monocytic lineage
ANOVA: Analysis of variance
DAPI: 4′,6-Diamidino-2-phenylindole
HBSS: Hanks Balanced Salt Solution
PBS: Phosphate buffered saline
PCR: Polymerase chain reaction
ELISA: Enzyme-linked immunosorbent assay
LPS: Lipopolysaccharide.
